# M1 Polarization Markers Are Upregulated in Basal-Like Breast Cancer Molecular Subtype and Associated With Favorable Patient Outcome

**DOI:** 10.3389/fimmu.2020.560074

**Published:** 2020-11-16

**Authors:** Mahmood Yaseen Hachim, Ibrahim Yaseen Hachim, Iman M. Talaat, Nada M. Yakout, Rifat Hamoudi

**Affiliations:** ^1^ College of Medicine, Mohammed Bin Rashid University of Medicine and Health Sciences, Dubai, United Arab Emirates; ^2^ Sharjah Institute for Medical Research, University of Sharjah, Sharjah, United Arab Emirates; ^3^ Clinical Sciences Department, College of Medicine, University of Sharjah, Sharjah, United Arab Emirates; ^4^ Pathology Department, Faculty of Medicine, Alexandria University, Alexandria, Egypt; ^5^ Division of Surgery and Interventional Science, University College London, London, United Kingdom

**Keywords:** basal like, breast cancer, macrophages, tumor infiltrated immune cells, transcriptomic

## Abstract

**Background:**

Breast cancer heterogeneity is an essential element that plays a role in the therapy response variability and the patient’s outcome. This highlights the need for more precise subtyping methods that focus not only on tumor cells but also investigate the profile of stromal cells as well as immune cells.

**Objectives:**

To mine publicly available transcriptomic breast cancer datasets and reanalyze their transcriptomic profiling using unsupervised clustering in order to identify novel subsets in molecular subtypes of breast cancer, then explore the stromal and immune cells profile in each subset using bioinformatics and systems immunology approaches.

**Materials and Methods:**

Transcriptomic data from 1,084 breast cancer patients obtained from The Cancer Genome Atlas (TCGA) database were extracted and subjected to unsupervised clustering using a recently described, multi-step algorithm called Iterative Clustering and Guide-gene Selection (ICGS). For each cluster, the stromal and immune profile was investigated using ESTIMATE and CIBERSORT analytical tool. Clinical outcomes and differentially expressed genes of the characterized clusters were identified and validated *in silico* and *in vitro* in a cohort of 80 breast cancer samples by immunohistochemistry.

**Results:**

Seven unique sub-clusters showed distinct molecular and clinical profiles between the well-known breast cancer subtypes. Those unsupervised clusters identified more homogenous subgroups in each of the classical subtypes with a different prognostic profile. Immune profiling of the identified clusters showed that while the classically activated macrophages (M1) are correlated with the more aggressive basal-like breast cancer subtype, the alternatively activated macrophages (M2) showed a higher level of infiltration in luminal A and luminal B subtypes. Indeed, patients with higher levels of M1 expression showed less advanced disease and better patient outcomes presented as prolonged overall survival. Moreover, the M1 high basal-like breast cancer group showed a higher expression of interferon-gamma induced chemokines and guanylate-binding proteins (GBPs) involved in immunity against microbes.

**Conclusion:**

Adding immune profiling using transcriptomic data can add precision for diagnosis and prognosis and can cluster patients according to the available modalities of therapy in a more personalized approach.

## Introduction

Breast cancer is one of the most common malignancies and accounts for most of the cancer-related deaths in women (1). Despite the significant advances in the diagnosis and the management of this disease, 20%–30% of patients with the early disease might end up with distant metastasis (2–4), which has no cure and is associated with poor prognosis (5). This is attributed to the disease heterogeneity and diversity at the molecular level, which play a role in the variability of clinical presentation and response to the standard treatment regimens (6).

Recently, technical developments in the transcriptomic and genomic profiling of tumors have improved our classification methods of breast cancer from the traditional clinicopathological classification into better and more distinct biological subtypes that showed distinct prognostic and therapeutic features (7–9). This includes luminal A, luminal B, HER-2 enriched, basal-like, and normal-like breast cancer (10). The adoption of such classification methods had led to significant improvement in patients’ stratification, drug selection, and outcome prediction. However, substantial heterogeneity is still observed within those groups in both genomic profiles and patient outcomes leading to unsatisfactory clinical results in many of the clinical trials (11).

One of the reasons proposed for the poor outcome is the fact that most of the classifications and analyses are focused on the tumor epithelial cells without deep investigation of the stromal microenvironment and its interaction with the malignant cells (12). In the past decades, efforts were made to investigate the molecular characterization of the tumor microenvironment (TME) and their role in modulating breast cancer cells’ behavior (13). Recently, tumor immune microenvironment (TIME) emerged as an essential factor that might explain the heterogeneity in breast cancer subtypes and their effect on prognosis and response to therapy (14).

The Cancer Genome Atlas (TCGA) Pan-Cancer studies investigating the immune cell subtypes in many malignancies revealed the inter and intra-cellular heterogeneity of the immune profile in breast cancer (15, 16). Similarly, the different breast cancer molecular subtypes showed a differential immune cell profile (17). Surprisingly, luminal A breast cancer subtype showed the most significant heterogeneity in their immune profile among the different breast cancers subtypes (15). Besides, the classification of luminal breast cancer according to the expression of immune-related genes showed better discrimination ability and prognostic stratification compared to the standard luminal A/B classification (18). Moreover, a comprehensive study was done by Lehmann et al, 2011 to investigate triple-negative breast cancer (TNBC) heterogeneity to identify clinically relevant subgroups that might provide the base of preclinical platforms for the development of more precise targeted therapeutic approaches. This study led to the discovery of six TNBC subtypes with distinct genomic, molecular, and biological features. These include two basal-like subgroups (BL1 and BL2), a luminal androgen receptor (LAR), a mesenchymal (M), a mesenchymal stem-like (MSL), and an immunomodulatory (IM) subgroup. Indeed, this report highlighted that IM subtype was highly enriched with immune cell signaling raising the query whether this enrichment is unique to the malignant cells or attributed to the stromal components including immune cell infiltrate (19). A subsequent report from the same group further refines TNBC molecular subtypes into only 4 tumor-specific subtypes and confirms that the IM and MSL subtypes were attributed to tumor-associated stromal cells as well as infiltrating lymphocytes (20).

All these together highlight the need for an integrated approach to stratify patients, taking into consideration the tumor cells’ characteristics as well as the TME, including the immune profile and stromal cells.

A recently described multi-step algorithm; Iterative Clustering and Guide-gene Selection (ICGS) identifies cell clusters through a five-step process: 1) PageRank-Down-sampling, 2) feature selection-ICGS2, 3) dimension reduction and clustering, 4) cluster refinement, and 5) cluster re-assignments using SVM (21) which showed promising results in resolving hidden cell population in complex datasets (22).

This study aims to use the publicly available transcriptomic database to stratify breast cancer into distinct molecular subtypes using unsupervised clustering, then to investigate the percentage of infiltrating immune cells and the status of their activation or polarization from their transcriptomic profile. Here, we used the unsupervised clustering methodology through ICGS to investigate breast cancer heterogeneity in 1084 breast cancer samples from TCGA (Pan-Cancer Atlas).

## Materials and Methods

### Breast Cancer Transcriptomics Data

The RNA seq data of 1,084 invasive breast cancer patients obtained from TCGA, Pan-Cancer Atlas, were retrieved from the cBioPortal online database “https://www.cbioportal.org/” (23). Details of the patients are listed in **Supplemental Table S1**.

### Unsupervised Clustering

**Figure 1:**
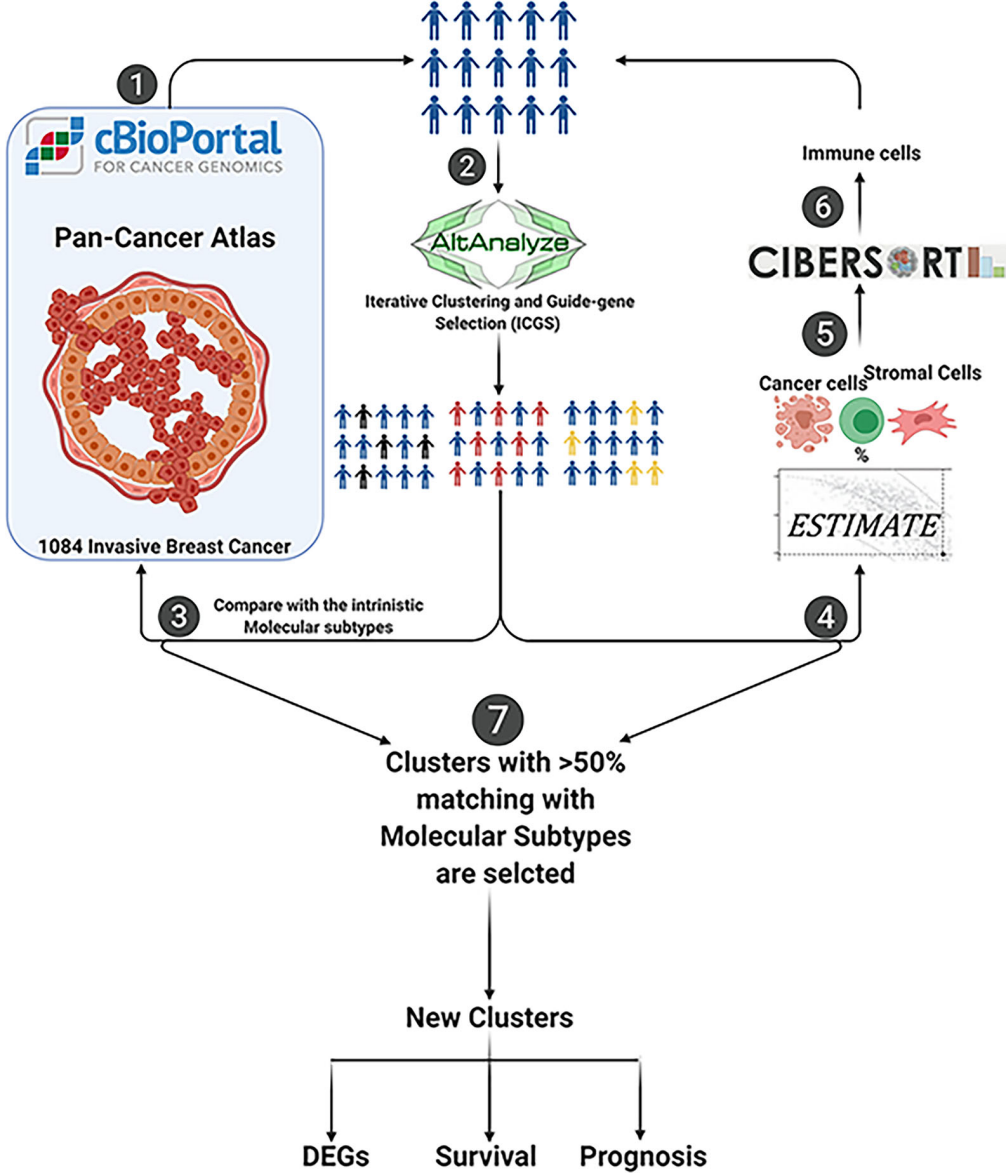
Flow chart of the bioinformatics methodology used.

Our initial approach was to perform unsupervised clustering of those samples into distinct sub-clusters based on their expression patterns independent of their clinicopathological features or intrinsic molecular subtypes. AltAnalyze tool was used for the unsupervised clustering of samples through ICGS2 (21, 22). The new clusters were compared with the clinicopathological data or intrinsic molecular subtypes, and those clusters that matched more than 50% of a given intrinsic molecular subtype were selected further. Samples that were clustered to the same pathological subtype were filtered for further analysis. The markers identification option in AltAnalyze tool listed the top differentially expressed genes (DEGs) between the groups. Genes with 2-fold change and adjusted p-value <0.05 were selected as cutoffs. The flow chart that represents the bioinformatics methodology used is shown in **Figure 1**.

### Gene Expression and Patient Outcome

We investigated the expression levels of each gene and their association with the patient outcome in TNBC samples using the publicly available Breast Cancer Gene-Expression Miner v4.0 (bc-GenExMiner v4.0) database (http://bcgenex.centregauducheau.fr/BC-GEM/GEM-Accueil.php?js=1) (24).

### Estimation of Stromal and Immune Cells Content in Tumor Tissues

To estimate the microenvironment percentage, ESTIMATE (Estimation of STromal and Immune cells in MAlignant Tumor tissues using Expression data) R Bioconductor package was used. ESTIMATE can predict tumor purity and the presence of infiltrating stromal/immune cells in tumor tissues using gene expression data (25).

### *In Silico* Prediction of the Immune Cell Infiltration

The raw mRNA expression of genes that are differentially expressed between the groups was used for *in silico* prediction of the immune cell infiltration using CIBERSORT analytical tool (26).

## Patients and Methods

### Patients

We used a tissue microarray of a patient cohort that consisted of 80 formalin-fixed paraffin-embedded breast cancer samples obtained from the Pathology Department, Faculty of Medicine, Alexandria University, Egypt. The clinicopathological data retrieved from the patients’ records, included age, grade, stage, therapeutic modalities, follow-up, and patient outcome. The cases were classified into the different molecular subtypes according to the hormonal receptors (ER, PR), HER-2, and Ki-67 status, in addition to CK5/6 expression, as previously described (27). Accordingly, 50 cases were classified as luminal B subtype, 20 cases as luminal A subtype, 7 cases as a basal-like triple-negative subtype, and only 3 cases as HER-2 enriched breast cancer subtype. The study was approved by the Research Ethics Committee of the Faculty of Medicine, Alexandria University, Alexandria, Egypt (approval number: 0103003).

### Immunohistochemistry

The slides were initially baked for 30 min in the oven at 55°C, this was followed by immersion in xylene for deparaffinization. The slides were then immersed in a serial dilution of alcohol for rehydration. Afterward, they were incubated with hydrogen peroxide block and were stained using two primary antibodies: Anti-Frizzled 9 antibody (ab61430) and Anti-NR2E1 (ab86276) (Abcam, Cambridge, United Kingdom). The UltraVision LP Detection System HRP Polymer & DAP Plus Chromogen (Thermo Fisher Scientific, Fremont CA) was used for visualization. The immunoreactivity of FZD9 was classified according to the intensity of four categories. Cases with no immunoreactivity were scored as 0, weak intensity cases were scored as +1, moderate-intensity as +2, and strong immunoreactivity was scored as +3. For statistical purposes, 0 and +1 staining were considered negative, while +2 and +3 were considered positive. For NR2E1, the cases were classified as negative if there was no evidence of immunoreactivity and positive when a positive staining pattern was interpreted.

## Results

### Unsupervised Clustering of Breast Cancer Samples Revealed the Presence of Seven Breast Cancer Sub-Clusters With Distinct Clinicopathological Features

**Figure 2:**
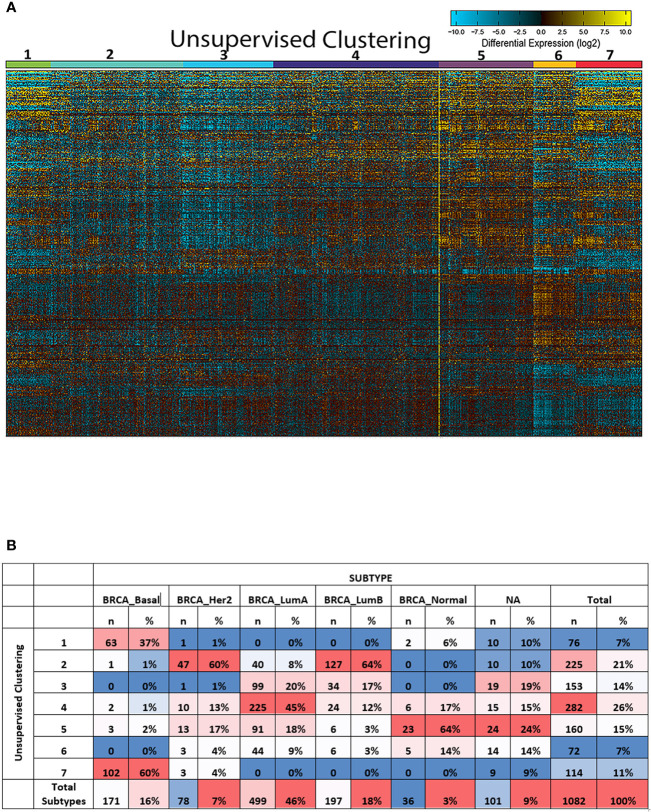
Unsupervised clustering of breast cancer subtypes revealed the presence of seven distinct sub-clusters. **(A)** Unsupervised clustering of the 1,084 breast cancer samples obtained from TCGA using ICGS2 option in the AltAnalyze tool. It showed the hierarchical cosine Euclidean option. **(B)** The distribution of the severe clusters in luminal A, luminal B, and basal-like subtypes and showing the representative groups that match more than 50% of the total patients in that molecular subtype.

**Figure 3:**
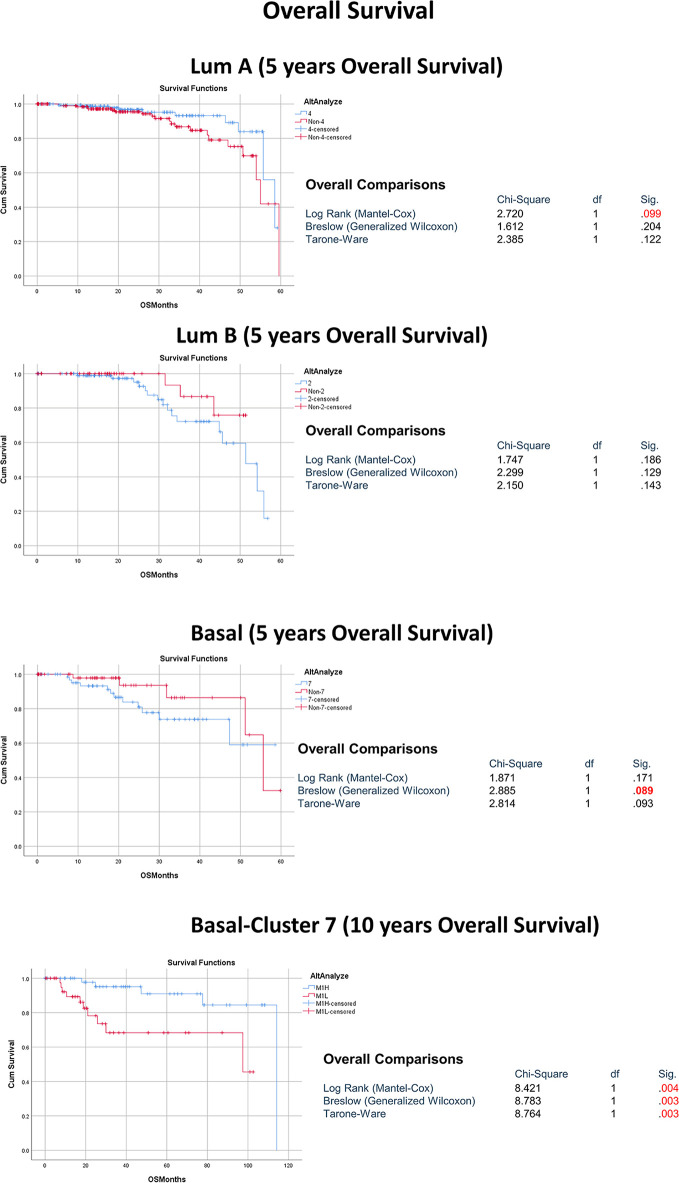
The association between the dominant sub-cluster within each molecular subtype and patient outcome.

Our in-silico approach revealed the presence of seven breast cancer sub-clusters that showed distinct molecular and genetic profiles (**Figure 2A**). Next, we investigated their association with the well-known intrinsic breast cancer subtypes. Interestingly, our analysis revealed a variable distribution of those sub-clusters within each breast cancer subtype (**Figures 2A, B**). At least one cluster represents the majority of cases from each molecular subtype. For example, cluster 4 was the dominant cluster in luminal A breast cancer samples. Similarly, cluster 2 was dominant in HER-2 tumors. Interestingly, the same cluster was the dominant cluster in luminal B tumors. In contrast, cluster 7 was the predominant cluster in the basal-like breast cancer subtype. The luminal A subgroup was the most heterogeneous with 45% of cases falling into cluster 4, 19.8% in cluster 3, 18.2% in cluster 5, and 8.8% falling into cluster 6. In comparison, the basal-like subgroup showed the least heterogeneity with samples falling mainly in cluster 7 (59.6%) and cluster 1 (36.8%). Further analysis revealed that different sub-clusters within each intrinsic subtype showed distinct clinical and survival features compared to other sub-clusters (**Figure 3**).

### Breast Cancer Sub-Clusters Representative of Intrinsic Breast Cancer Molecular Subtypes Showed a Distinct Genetic and Molecular Profile

Having proved that each intrinsic breast cancer subtype represents a group of distinct sub-clusters, further *in-silico* analysis with the aim of investigating the genetic and molecular profile of the main sub-clusters that represent each molecular subtype was performed. Indeed, cluster 4 was selected as representative for luminal A tumors (n=225, 45%), cluster 2 for luminal B (n=127, 64%) and cluster 7 for basal-like subtypes (n=102, 60%) (**Figure 2B**). Interestingly, our approach revealed a group of novel genes that can differentiate basal-like breast cancer from other breast cancer subtypes (**Table S2**), including the *POU5F1* gene (*OCT4*), which was previously found to be associated with TNBC and linked to cancer stem cells and worse patient outcome. Most of the other genes were not fully investigated and their role in TNBC cancer is not yet known. Similarly, a seven-gene signature was found to be able to differentiate luminal A tumors from luminal B and basal-like breast cancer subtypes. For luminal B, two genes were found to be able to differentiate the luminal B breast cancer subtype from both luminal A and basal-like breast cancer subtype. Our results here identified novel top differential genes that can clearly differentiate between the different breast cancer subtypes.

### Eight of Our Identified Top Differential Genes From the Basal-Like Breast Cancer Were Confirmed to Be Upregulated in Samples From TNBC Patients and Their Expression to Be Associated With Worse Outcome

**Figure 4:**
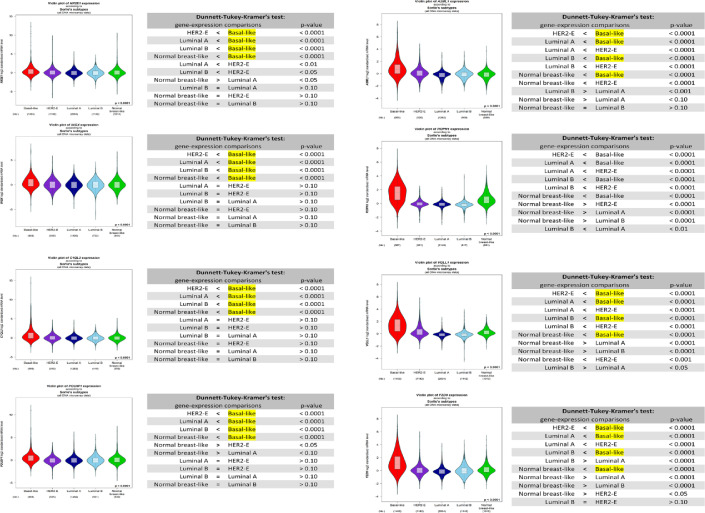
Box plot of NR2E1, INGX, C1QL2, POU5F1, A2ML1, ROPN1, VGLL1, FZD9 expression in different breast cancer subtypes using Breast Cancer Gene-Expression Miner v4.0 database.

Next, we focussed on the gene signature that was able to differentiate between TNBC and non-TNBC subtypes. For that reason, we used another in-silico tool to investigate the expression levels of each of those genes and their association with the patient outcome in TNBC samples in the publicly available Breast Cancer Gene-Expression Miner v4.0 (bc-GenExMiner v4.0) database. Out of the 25 gene signature, eight genes were shown to be upregulated in TNBC samples compared to other molecular subtypes and to be associated with poor prognosis in those patients. The genes included *NR2E1, INGX, C1QL2, POU5F1, A2ML1, ROPN1, VGLL1, FZD9* (**Figure 4**, **Supplemental Figure 1**).

### *NR2E1* and *FZD9* Were Confirmed to Be Upregulated in Basal-Like TNBC Samples From Our Patient Cohort Consisting of 80 Breast Cancer Specimens

**Figure 5:**
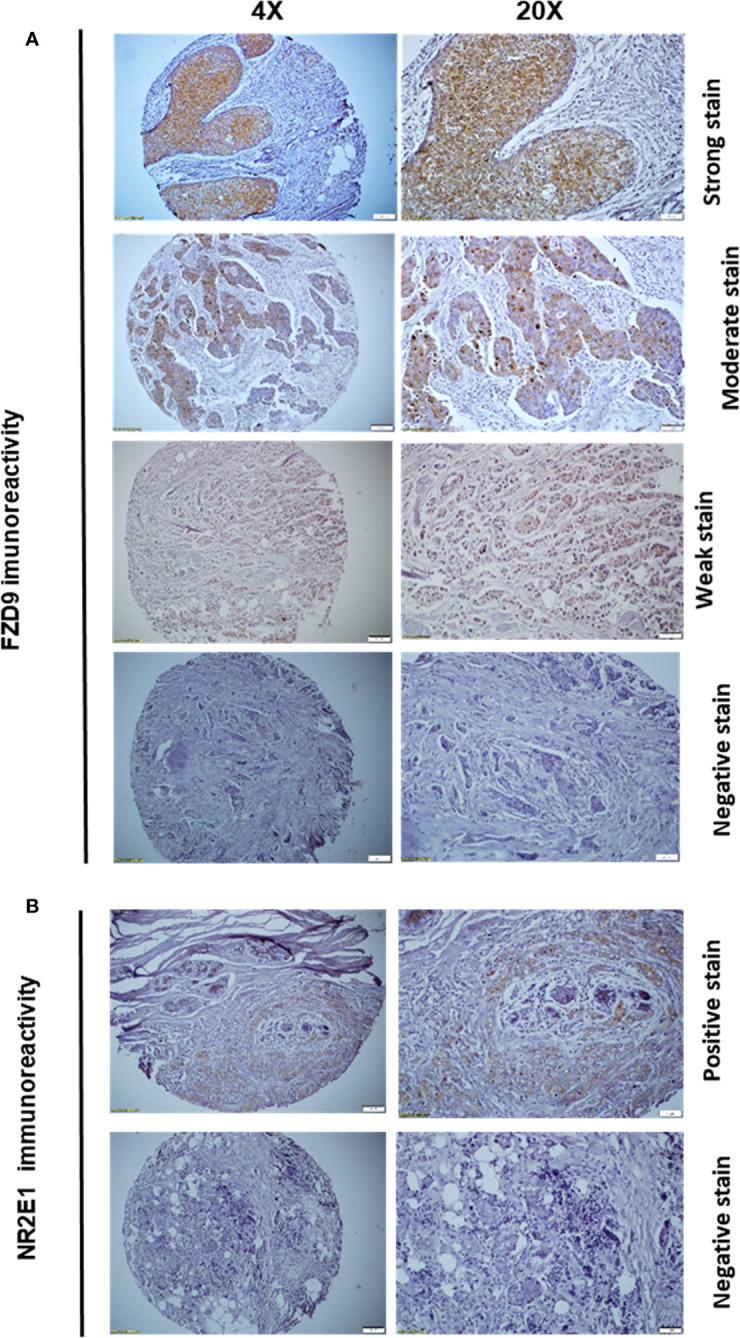
FZD9 and NR2E1 immunoreactivity in our patient cohort that consists of 80 breast cancer patients **(A)** Representative images of strong, moderate, weak as well as negative FZD9 immunoreactivity. **(B)** Representative images of positive and negative NR2E1 immunoreactivity.

As a proof of concept, and to confirm the accuracy of our shortlisted TNBC gene signature, we investigated the protein expression levels of two genes (*NR2E1 and FZD9*) using immunohistochemistry (IHC) in our patient cohort (**Figure 5**). The cohort consisted of 80 breast cancer samples from different molecular subtypes, 50 cases of luminal B, 20 cases of luminal A, 7 cases of basal-like triple-negative, and only 3 cases of HER-2 enriched.

The overall expression of *FZD9* in the cohort was limited to only 27.5% of all samples (**Table 1**) (**Figure 5A**). This can be explained by the fact that around 70% of the tumor samples in our cohort were confined to either luminal A or B breast cancer subtypes. Besides, our results showed no association between *FZD9* IHC expression and tumor size, LN status, or tumor stage (**Table 1**). While it does not reach a statistical significance, a significant trend was observed between FZD9 expression and tumor grade. Tumors with moderate to poor differentiation (grade II & III) showed around three times *FZD9* levels (29.82% and 27.27%, respectively) compared to well-differentiated tumors that showed positivity in only 9% of the cases (**Table 1**). Interestingly, the classification of samples according to their molecular subtypes revealed a significantly higher expression of *FZD9* levels in the basal-like TNBC subtype (71.42%) compared to samples from other non-TNBC samples (23.28%) (P=0.0224). Finally, our results showed that patients with worse outcomes and a decline in the survival expressed higher levels of *FZD9* (35.7%) compared to patients who had a better outcome with prolonged survival (*FZD9* positivity in only 21.5% of the samples) (**Table 1**).

**Table 1:** The association between FZD9 and NR2E1 immunoreactivity in different clinicopathological parameters in our patient cohort that consists of 80 breast cancer patients.

Clinicopathological Parameters	FZD expression			P
	Positive	Negative	%	P value
**Grade**				
Grade 1	1	10	9.09%	P=0.36
Grade 2	17	40	29.82%	
Grade 3	3	8	27.27%	
**Tumor size**				
<5	13	31	32.5%	P=0.74
>5	7	20	26.92%	
**LN status**				
LN negative	4	10	28.57%	P=0.97
LN positive	16	41	28.57%	
**Stage**				
Stage 1,2	9	21	30%	P=0.72
Stage 3, 4	11	30	26.82%	
**Molecular subtype**				
Non-TNBC	17	56	23.28%	**P=0.0224**
TNBC	5	2	71.42%	
**Patient outcome**				
Alive	11	40	21.56%	P=0.17
Died	10	18	35.71%	
**Clinicopathological Parameters**	**NR2E1 expression**			**P value**
	**Positive**	**Negative**	**%**	**P value**
**Grade**				
Grade 1	2	9	18.18%	P= 0.81
Grade 2	11	46	19.29%	
Grade 3	3	8	27.27%	
Tumor size				
<5	10	34	22.72%	P= 0.96
>5	6	21	22.22%	
**LN status**				
LN negative	4	11	26.67%	P=0.97
LN positive	12	43	22.22%	
Stage				
Stage 1,2	7	23	23.33%	P= 0.89
Stage 3, 4	9	32	21.95%	
**Molecular subtype**				
Non-TNBC	12	60	16.67%	**P=0.04**
TNBC	4	3	57.14%	
**Patient outcome**				
Alive	9	41	18%	P= 0.46
Died	7	21	25%	

The bold values mean significant. p < 0.05.

Similarly, the *NR2E1* showed the same trend with no significant association with most of the clinicopathological parameters (**Table 1**) (**Figure 5B**). However, basal-like TNBC samples showed significantly higher levels of *NR2E1* (57.14%) compared to the non-TNBC samples (%16.67) (P=0.04). Also, while it was not significant, patients with higher grade (grade III) showed higher levels of *NR2E1* expression compared to grade II (19.29%) and grade I (18.18%). Moreover, patients with poor overall survival showed a higher level of *NR2E1* (25%) compared to patients with better overall survival (18%) (**Table 1**). The results obtained from our IHC panel for both *FZD9* and NR2E1 demonstrated the sensitivity of our shortlisted genes in discriminating between basal-like TNBC samples and other breast cancer molecular subtypes.

### Breast Cancer Sub-Clusters Representative of Intrinsic Breast Cancer Molecular Subtypes Showed a Distinct Stromal and Immune Cell Profile Including Macrophages 0, 1, and 2

**Figure 6:**
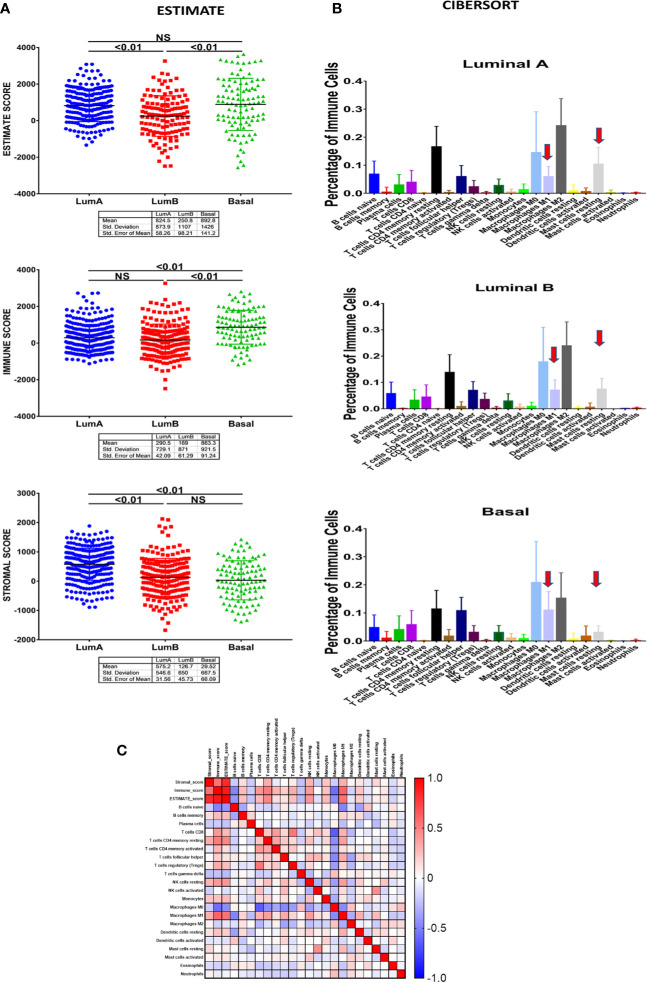
The cellular and stromal profile of clusters representative of luminal A, B, and basal-like breast cancer subtypes. **(A)** Estimation of Stromal and Immune cells profile in clusters representative of luminal A, B, and basal-like breast cancer subtypes using Expression data (ESTIMATE) tool. **(B)** The percentage of immune cell infiltration in basal versus luminal A and B breast cancer cells as predicted by CIBERSORT analytical tool. **(C)** Non-parametric Pearson correlation matrix for immune cells showing different correlation of M1 and M2 to other infiltrating immune cells.

Due to the increasing importance of the microenvironment in breast cancer heterogeneity and determining cancer cells’ behavior, we next investigated whether our identified clusters have distinct infiltrating stromal cells in addition to immune cells profile. To achieve this we used a different *in-silico* tool; the Estimation of STromal and Immune cells in MAlignant Tumours using Expression data’ (ESTIMATE) tool, which is a method that depends on the analysis of gene expression signature to identify the stromal and immune cells fractions in a given tumor sample (**Figure 6A**). Our results showed a statistically significant difference in tumor purity in terms of the immune and stromal score. The immune score of the basal-like subtype (863.3 ± 91.24) was statistically higher compared to luminal A (290.5 ± 42.09) and luminal B (169 ± 61.29) subtypes (P<0.001) (**Figure 6A**). Also, investigating the percentage of immune cell infiltration in our clusters representing basal versus luminal A and B breast cancer as predicted by CIBERSORT analytical tool (**Figure 6B**) revealed macrophages M0 and M1 to be significantly higher in the basal-like subtype compared to luminal A and luminal B (p<0.001). In contrast, macrophages M2 was significantly higher in the luminal A (p<0.001) and luminal B (p<0.001) subtypes compared to the basal-like subtype. Details of CIBERSORT results are listed in **Supplementary Table S3**.

This clearly demonstrated that our unsupervised clustering was not only able to distinguish cancer cells in different breast cancer subtypes but also differentiate the microenvironment profile within those molecular subtypes.

### M1 and M2 Showed Different Correlation With Other Immune Infiltrating Cells

Next, and for a better understanding of the role of distinct M1 and M2 infiltrate on modulating the immune response and its possible effect on patient outcome, we investigated the non-parametric Pearson correlation matrix between M1 and M2 and its association with a large panel of other immune cells (**Figure 6C**). Interestingly, while our results revealed a negative correlation between M2 expression and most of the other immune cells, M1 expression showed a positive correlation with most of the other immune cells, including CD8+ T cells as well as NK cells, known to have a pivotal role in the host anti-tumor response.

### Macrophage 1 Level Can Identify Two Basal-Like Subgroups That Showed Distinct Genomic as Well as Clinicopathological Features With M1 High Subgroup to Be Associated With a Better Overall and Disease-Free Survival

**Figure 7:**
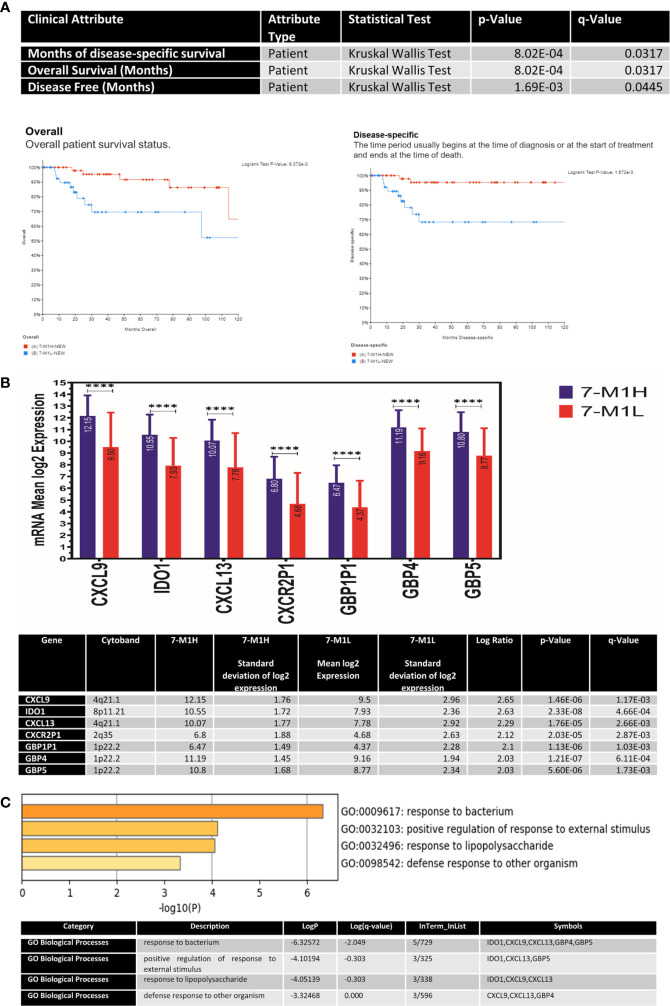
The genetic and molecular profile of M1H basal-like breast cancer subtype compared to the M1L group **(A)** The association between M1H and M1L basal-like breast cancer subtypes and patient outcome presented as overall survival (OV) and disease-free survival (DFS). **(B)** The expression levels of key M1 phenotype markers including specific cytokines and chemokines (CXCL9, IDO1, CXCL13, CXCR2P1, GBP1P1, GBP4and) in M1H and M1L basal-like breast cancer subtypes. **(C)** Top pathways enriched in M1H basal-like breast cancer subtype. ****p < 0.01.

Next, we investigated if our observed distinct immune profile, including the tumoricidal classically activated M1, affects the clinical course and outcome of the basal-like breast cancer patients. To achieve this, the basal-like breast cancer samples were divided according to their macrophage percentage as per the CIBERSORT immune cells prediction using a transcriptome profile. M1-M0 to M2-M0 ratio was used to define two basal-like breast cancer groups: M1 high (M1H) if the ratio is above 0 and M1 low (M1L) if the ratio is less than 0 (**Table S3**). Comparing all the clinical data of the M1H and M1L basal-like breast cancer patients, the only significant difference shown was in the overall and disease-free survival. The M1H basal-like overall (q-value=0.0317) and disease-free survival (q-value=0.0445) are significantly better than M1L basal-like group (**Figure 7A**).

### M1H Basal-Like Group Showed a Distinct Antibacterial Immune-Related Transcriptomic Profile Than the M1L Group

Indeed, there was no significant difference in the rate of mutations between the two groups, and no gene copy number changes; however, there was a significant difference in the gene expression as measured by RNAseq. Our results showed clearly that basal-like breast cancer with M1H and M1L subgroups represent two different entities in their genomic characteristics. Besides, our results also revealed that the M1H subgroup to be enriched with 1. specific cytokines and chemokines (*CXCL9, CXCL13, CXCR2P1*), 2. the guanylate-binding protein family GTPases which is induced by interferon-gamma (IFN-γ) to protect against microbial and viral pathogens (*GBP1P1, GBP4, and GBP5I*) and 3. indoleamine 2,3-dioxygenase 1 (*IDO1*) which is an immune modulator enzyme and has a bactericidal activity through direct anti-pathogen mechanisms *via* depletion of tryptophan. All of these showed a 2-fold change with a p-value <0.05 (**Figure 7B**).

The identified genes were enriched in antibacterial immune responses related pathways including response to the bacterium, positive response regulation to an external stimulus, response to lipopolysaccharide, and defense response to other organisms (**Figure 7C**).

All these together highlight the importance of our unsupervised clustering in the identification of more precise breast cancer subtypes with distinct malignant cells and microenvironment profile.

## Discussion

In this study, we tried to use a comprehensive *in silico* approach to dissect the inter and intra-tumoral heterogeneity of different breast cancer subtypes and their impact on cancer cells’ behavior and patient outcome. We pooled data of 1,084 breast cancer patients from different breast cancer subtypes using TCGA cohorts. Our analysis was done through an unsupervised single-cell population identification method that revealed a unique list of genes that were able to clearly differentiate between different breast cancer molecular subtypes.

Due to its poor prognosis and lack of targeted therapy, we further investigated the top differential genes in the overly aggressive TNBC subtypes. Our analysis using different independent, publicly available databases confirm that eight of our shortlisted top differential genes were upregulated in TNBC and their expression to be associated with worse patient outcome. Except for the *POU5F1* gene (*OCT4*), which is a cancer stem cell marker and its expression was found to be associated with worse outcome (28), the role of the other seven genes in TNBC tumorigenesis, as well as their prognostic significance, is not yet well identified.

As a proof of concept, we confirmed the clinical significance and prognostic value of two of the eight shortlisted genes (*NR2E1, FZD9*) belonging to this gene signature in our patient cohort consisting of 80 breast cancer patients. Both genes were able to discriminate TNBC samples from other non-TNBC subtypes independent of their clinicopathological parameters. This indicates the accuracy and sensitivity of our filtration method and bioinformatic approach. However, further studies with a larger number of patients are still needed in the future to confirm the clinical benefits of using those genes as predictive and prognostic markers.

Interestingly, *NR2E1* was recently found to be upregulated in ERα-negative breast cancer and to play a role in breast cancer cell growth and invasion and was suggested as a possible candidate for therapeutic targeting (29). Similarly, *FZD9*, which belongs to frizzled receptors (*FZD*s) family that are G protein-coupled receptors essential for WNT signaling pathway, was also found recently to be upregulated in a group of cancers including the highly aggressive astrocytoma as well as osteosarcoma, and its knockdown was shown to reduce cell proliferation and motility in hepatocellular as well as hepatoblastoma cell lines (30, 31).

While our results clearly demonstrated the clinical significance of our gene signature, further studies should be performed to investigate the other candidates before its implementation in the clinical practice.

Another important finding in our study is the ability of our stratification method not only to detect the cancer cells heterogeneity but also to detect heterogeneity in the microenvironment profile, including the stroma as well as the immune cell profile. This was evident in our findings; the immune score was statistically higher in the basal-like subtype compared to both luminal A & B subtypes. Indeed, our results provide evidence that the enrichment of tissues from IM subtype of TNBC patients with immune cell signaling and pathways observed by Lehmann et al, 2011 was not only due to epithelial tumor cells but also due to the difference in the tumor microenvironment including the immune cells as well as the stromal components surrounding the tumor (19). Moreover, our findings go with the previous report that showed a significant association between poor classical clinicopathological parameters, including ER, PR negativity, LN involvement, poorly differentiated tumors, and the absolute Immunoscore, which reflects the total tumor-infiltrating immune cells (32). The same report also showed tumor-infiltrating lymphocytes (TILs) to be higher in the more aggressive HER-2 as well as basal-like breast cancer types compared to the less aggressive luminal A & B subtypes (32).

Interestingly, our signature was also able to identify a distinct immune cell profile, including macrophages (M0, M1, and M2) in the different breast cancer subtypes. Our findings revealed that stratification of basal-like TNBC samples according to macrophage M1 level was able to identify two basal-like subgroups with distinct genomic as well as clinicopathological features in addition to distinct patient outcomes. This highlighted the need for such a signature that not only detect cancer cell heterogeneity but also able to identify TME variation, including the immune cells that recently became an important candidate for new therapeutic options, including immunotherapy.

Besides, our results also showed preliminary evidence on the molecular basis of the beneficial effect and favorable outcome of high M1 expression in basal-like TNBC. Indeed, we have found that the M1H subgroup is enriched with M1 phenotype markers that were enriched in antibacterial immune responses related pathways. Some of those pro-inflammatory markers were previously found to be involved in leukocyte trafficking, including integrin activation and chemotactic migration, and their expression was found to promote M1 polarization and predict response to therapy as well as favorable patients outcome (33, 34). This goes with the anti-tumorigenic and pro-inflammatory effects proposed for M1-like macrophages. This was reflected in the prolonged overall survival observed in this subgroup compared with the M1L subgroup.

**Figure 8:**
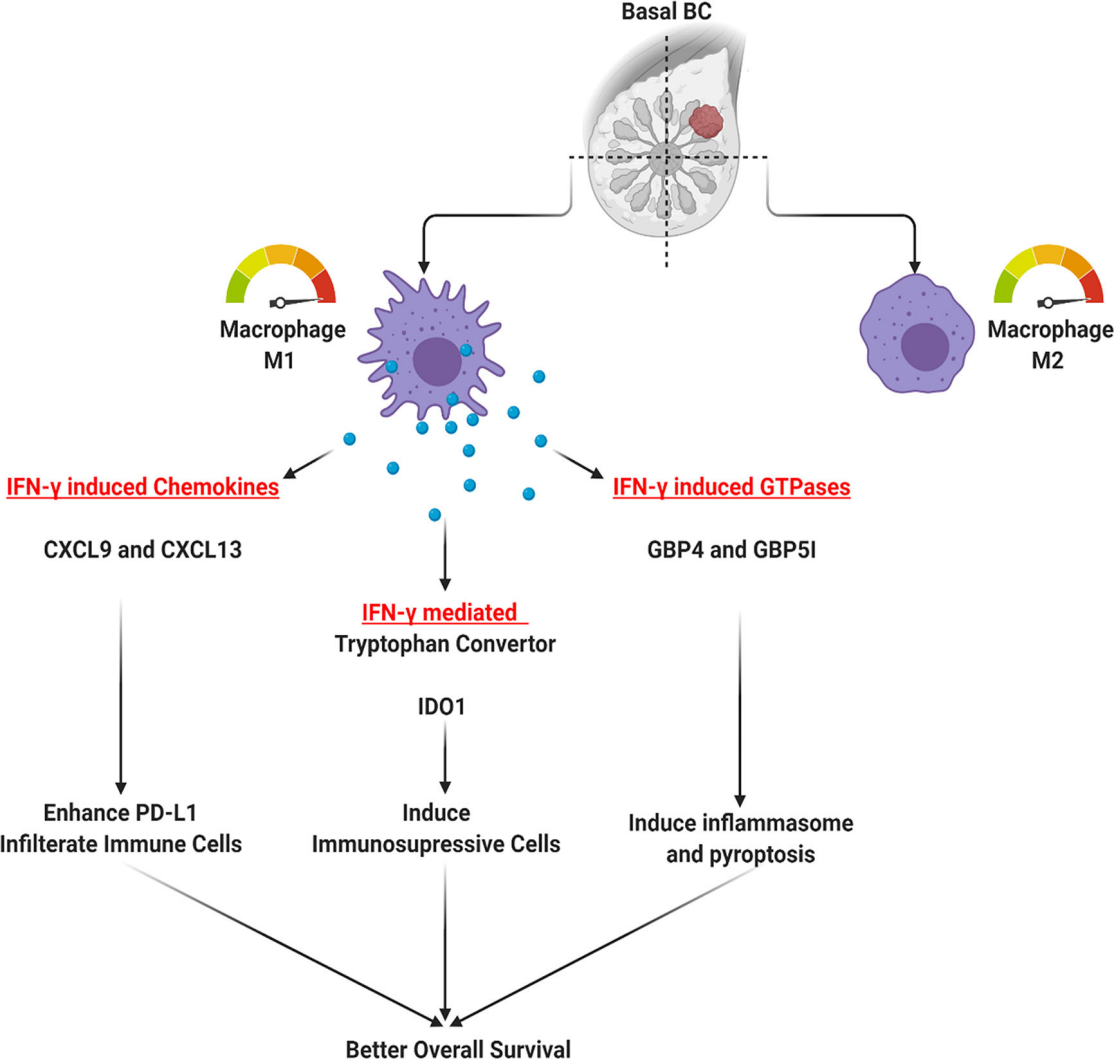
Possible mechanisms through which M1 might mediate the favorable outcome in basal-like triple-negative breast cancer (TNBC) patients.

As shown in **Figure 8**, we proposed three mechanisms that might explain the favorable outcome of the M1H subgroup; all of which are mediated through IFN-γ. The first mechanism is mediated through IFN-γ induced chemokines (*CXCL9 and CXCL13*). Indeed, chemokine *CXCL9* is induced by IFN-γ to mediate lymphocytic infiltration to the focal sites thus suppressing tumor growth (35), in addition, *CXCL9* was found to be significantly associated with increased pathologic complete response rate (pCR) in breast cancer (36) and prolonged disease-free and overall survival in patients with the triple-negative disease (37).

Moreover, the response rates to the immune checkpoint blockade (ICB); anti-PD-1/anti-CTLA-4 in breast cancer was found to have a direct correlation with the extent of tumor immune infiltrate, which is correlated with upregulated macrophages derived CXCR3 ligands, *CXCL9*, *and CXCL10* (38). This *CXCL9/10/11-CXCR3* axis activation can lead to upregulated expression of the immunosuppressor programmed death-ligand 1 (PD-L1) by activating the STAT and PI3K-Akt signaling pathways that play an important role in cancer treatment (39).

On the other hand, chemokine C-X-C motif ligand 13 (*CXCL13*) plays a fundamental role through *CXCL13: CXCR5* axis during inflammatory, infectious and immune responses by orchestrating lymphocyte infiltration within the TME (40). As in the case of *CXCL9*, IRF5 (interferon regulatory factor 5) can bind to the promoter of *CXCL13* and directly regulate its expression in mammary epithelial tumor cells leading to the infiltration of CD19+CXCR5+ B-cell and CD4+CXCR5+ T-cell to the tumor (41). But opposite to *CXCL9*, high *CXCL13* was associated with improved outcomes in the luminal-human epidermal growth factor receptor two subtypes (37).

Another mechanism that might explain the M1H subgroup favorable outcome is through the other top DEGs between M1H and M1L subgroups, including the IFN-γ induced GTPases, known to protect against microbial and viral pathogens (*GBP4* and *GBP5I*). *GBP* genes could act as protective factors in host defense by controlling infection and autoimmunity (42). Infection-driven IFN maintains GBP expression in murine and human macrophages needed to restrict intracellular pathogens (43) through the activation of caspase‐1 containing inflammasome complexes or caspase‐4, which triggers pyroptosis (44). Mechanistically, recruited neutrophils mediate bacterial clearance through the Gbp4 inflammasome-dependent biosynthesis of prostaglandin D2 (45). In breast cancer, inflammasome was linked to myeloid-derived suppressor cells (MDSCs) and tumor-associated macrophages (TAMs) infiltration providing an inflammatory microenvironment (46).

Our results also identified a major bactericidal activity and immune modulator enzyme: IDO1, which was specifically upregulated in M1H group. Indeed, IDO1 was found to be one of the immune checkpoint proteins involved in cancer immune escape (47). However, and similar to our findings, a recent report with comprehensive genomic analysis identifying novel TNBC subtypes, also recognizes IDO1 as one of the most highly expressed genes in an immune-activated basal-like TNBC subtype with high TILs density, suggestive of active immune reaction (47, 48). Moreover, it was also found to be significantly up‐regulated in basal-like breast cancer subtype than the other subtypes and showed better survival prognosis as it is involved in interferon-gamma response and PD‐L1 positivity (49).

Overall, our results highlight the importance of using a combined approach that consists of high-throughput genomic technologies and unsupervised single-cell clustering methods in exploring breast cancer heterogeneity. This approach might be essential not only to understand the intratumoral heterogeneity but also for the discovery of more clinically relevant patients’ subpopulations and the discovery of new potential biomarkers and therapeutic targets that are not restricted to parenchymal cells but extend to the stromal and immune cell infiltrate. Such an approach might help in a more personalized and better patients response to different therapeutic options, including chemotherapy, radiotherapy, as well as targeted therapy.

## Conclusion

Our approach was able to identify discrete sub-clusters within breast cancer subtypes with a distinct molecular and clinical profile. Those sub-clusters not only identified heterogeneity between the different breast cancer subtypes but also highlighted intra-subtype heterogeneity. Moreover, our clustering methods were able to differentiate breast cancer samples not only according to the cancer cells profile but also according to the TME, including both stromal as well as immune cell profiles. Adding immune profiling through transcriptomic data can increase precision for diagnosis and prognosis of breast cancer patients and can categorize patients according to the available therapeutic modalities in a more personalized approach.

## Data Availability Statement

The original contributions presented in the study are included in the article/**Supplementary Material**. Further inquiries can be directed to the corresponding authors.

## Ethics Statement

The studies involving human participants were reviewed and approved by Ethics Committee of the Faculty of Medicine, Alexandria University (Alexandria, Egypt), with a registration number (0103003). The patients/participants provided their written informed consent to participate in this study.

## Author Contributions

Conceptualization: MH and IH. Methodology: MH and IH. Validation: MH, IH, and IT. Formal analysis: MH and IH. Investigation: MH and IH. Resources: RH and IT. Data curation: MH and IH. Writing—original draft preparation: MH and IH. Writing—review and editing: MH, IH, IT, NY, and RH. Supervision: IT and RH. Project administration: IT and RH. Funding acquisition: IT and RH. All authors contributed to the article and approved the submitted version.

## Funding

This project was funded by the Al-Jalila Foundation (Grant No: AJF201741) and the University of Sharjah research funding program, (Project No: 1901090255-P).

## Conflict of Interest

The authors declare that the research was conducted in the absence of any commercial or financial relationships that could be construed as a potential conflict of interest. 

## Supplementary Material

Supplementary Figure 1 Kaplan-Meier survival analysis of NR2E1, INGX, C1QL2, POU5F1, A2ML1, ROPN1, VGLL1, FZD9 using Breast Cancer Gene-Expression Miner v4.0 database.
