# Supplementary figures and images for: M1 Polarization Markers Are Upregulated in Basal-Like Breast Cancer Molecular Subtype and Associated With Favorable Patient Outcome
Source: Front Immunol. 2020 Nov 16;11:560074. doi: 10.3389/fimmu.2020.560074 (PMC7701279; doi:10.3389/fimmu.2020.560074)

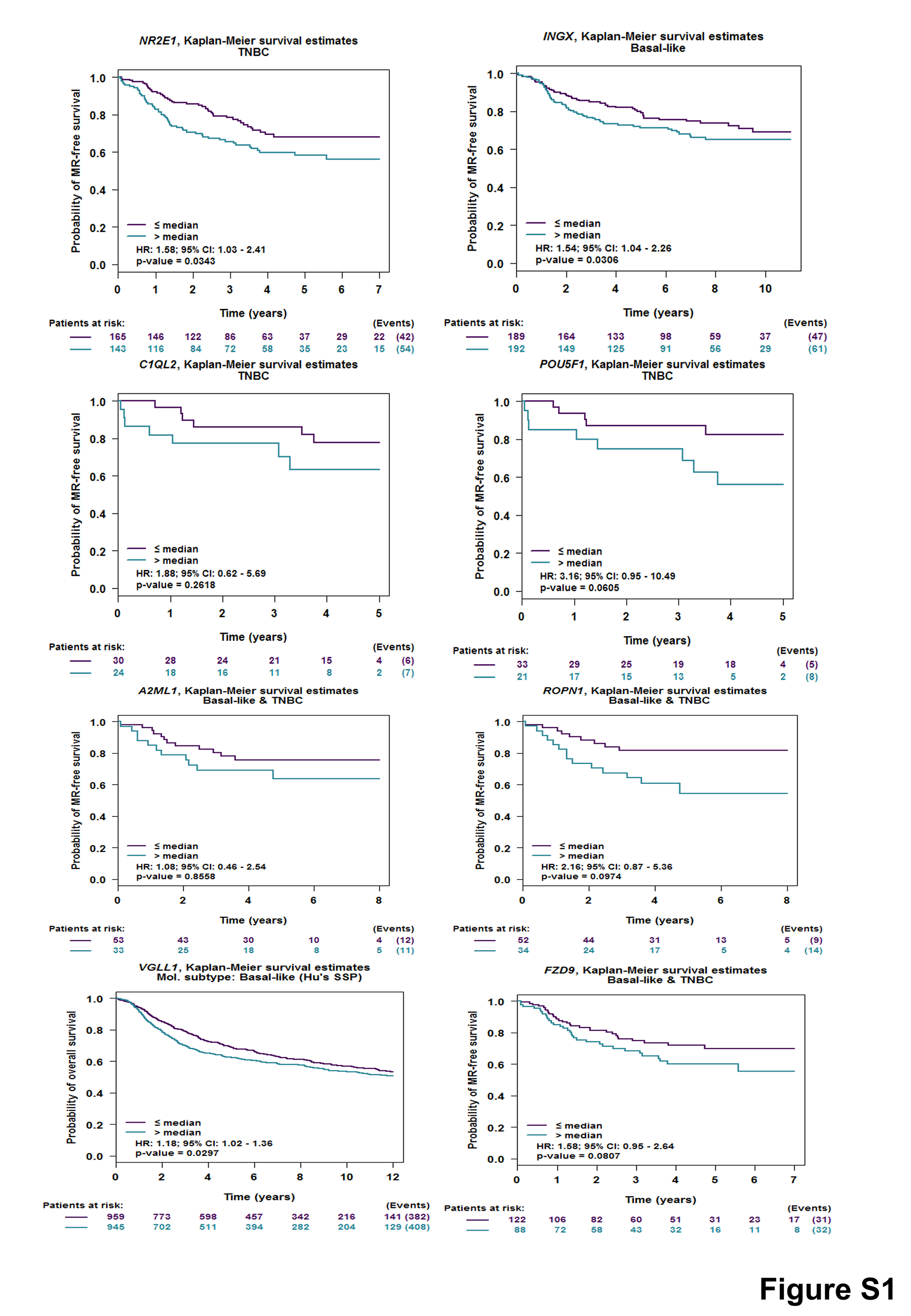

Supplement: Supplementary Figure 1 — Kaplan-Meier survival analysis of NR2E1, INGX, C1QL2, POU5F1, A2ML1, ROPN1, VGLL1, FZD9 using Breast Cancer Gene-Expression Miner v4.0 database. [file Image_1.tif]
