# Supplementary material for: M1 Polarization Markers Are Upregulated in Basal-Like Breast Cancer Molecular Subtype and Associated With Favorable Patient Outcome
Source: Front Immunol. 2020 Nov 16;11:560074. doi: 10.3389/fimmu.2020.560074 (PMC7701279; doi:10.3389/fimmu.2020.560074)
Supplement: Supplementary file 2 [file Table_1.docx]

Supplementary

Table S1: Details of the 1084 invasive breast cancer patients obtained from TCGA, PanCancer Atlas, were retrieved from cBioPortal online database <https://www.cbioportal.org/>

A. Cancer Type Detailed

| **Cancer Type Detailed** | **Number** | **%** |
| --- | --- | --- |
| Breast Invasive Lobular Carcinoma | 201 | 19% |
| Breast Invasive Ductal Carcinoma | 780 | 72% |
| Breast Invasive Carcinoma (NOS) | 77 | 7% |
| Breast Invasive Mixed Mucinous Carcinoma | 17 | 2% |
| Metaplastic Breast Cancer | 8 | 1% |
| Invasive Breast Carcinoma | 1 | 0% |
| Total | 1084 | 100% |

B.Molecular Subtypes

| **Subtype** | **Number** | **%** |
| --- | --- | --- |
| **BRCA_LumA** | **499** | **46%** |
| **BRCA_Her2** | **78** | **7%** |
| **BRCA_LumB** | **197** | **18%** |
| **BRCA_Normal** | **36** | **3%** |
| **BRCA_Basal** | **171** | **16%** |
| **Others** | **274** | **25%** |
| **Total** | 1084 | 100% |

Table S2: Details of the identified DEGs between Basal, Luminal A and Luminal B

| Gene | marker-in | BASAL | LumA | LumB |
| --- | --- | --- | --- | --- |
| 1. CXorf61 | BASAL | 5.106201 | 0.116733 | 0.202592 |
| 1. CXorf49B | BASAL | 4.857335 | 0.218984 | 0.277427 |
| 1. NR2E1 | BASAL | 2.91011 | 0.296015 | 0.307527 |
| 1. HORMAD1 | BASAL | 6.195274 | 0.83295 | 0.784833 |
| 1. OLAH | BASAL | 3.397809 | 0.504662 | 0.275395 |
| 1. INGX | BASAL | 1.863805 | 0.287755 | 0.34698 |
| 1. ART3 | BASAL | 7.277275 | 1.167184 | 0.922769 |
| 1. C1QL2 | BASAL | 4.644245 | 0.860753 | 0.347974 |
| 1. LEMD1 | BASAL | 4.898313 | 0.938986 | 0.54074 |
| 1. GABBR2 | BASAL | 5.712272 | 1.137985 | 1.288945 |
| 1. MATN4 | BASAL | 3.879345 | 0.784809 | 0.82273 |
| 1. SLC26A9 | BASAL | 5.303361 | 1.084642 | 1.126662 |
| 1. A2ML1 | BASAL | 8.842424 | 1.856431 | 2.814754 |
| 1. AMY1A | BASAL | 6.301817 | 1.50242 | 1.002383 |
| 1. RHCG | BASAL | 6.158168 | 1.678112 | 1.331562 |
| 1. ROPN1 | BASAL | 7.583997 | 2.115256 | 0.942125 |
| 1. NCRNA00092 | BASAL | 4.404999 | 1.259552 | 0.77639 |
| 1. VGLL1 | BASAL | 8.823178 | 2.661397 | 1.667347 |
| 1. POU5F1 | BASAL | 3.781839 | 1.203501 | 1.425379 |
| 1. C9orf170 | BASAL | 3.325371 | 1.067622 | 0.514734 |
| 1. FZD9 | BASAL | 6.231108 | 2.106699 | 2.19347 |
| 1. FABP7 | BASAL | 7.905908 | 2.702688 | 1.091654 |
| 1. WNT6 | BASAL | 6.050611 | 2.149023 | 1.564108 |
| 1. ROPN1B | BASAL | 7.01033 | 2.577138 | 1.558265 |
| 1. CHODL | BASAL | 6.913248 | 2.786287 | 1.895462 |
| 1. LOC100128977 | LumA | 0.158153 | 3.770917 | 1.525915 |
| 1. SCN2B | LumA | 1.815336 | 4.724666 | 2.112369 |
| 1. ZBTB16 | LumA | 1.949293 | 5.345623 | 2.834728 |
| 1. SCN7A | LumA | 1.214267 | 5.358342 | 2.901372 |
| 1. ABCA8 | LumA | 3.937865 | 7.143696 | 4.307435 |
| 1. KERA | LumA | 1.702193 | 4.947761 | 3.042515 |
| 1. ANKRD30A | LumA | 1.313677 | 9.344966 | 6.084819 |
| 1. TEX19 | LumB | 1.273306 | 1.463603 | 3.458219 |
| 1. KIAA0319 | LumB | 2.863808 | 2.636319 | 4.695355 |

Table S3: Percentage of immune cells (Macrophages 0,1 and 2) infiltration in basal versus luminal A and B breast cancer as predicted by CIBERSORT analytical tool

|  | LumA-M0 | LumB-M0 | Basal-M0 | LumA-M1 | LumB-M1 | Basal-M1 | LumA-M2 | LumB-M2 | Basal-M2 |
| --- | --- | --- | --- | --- | --- | --- | --- | --- | --- |
| Number of values | 225 | 127 | 102 | 225 | 127 | 102 | 225 | 127 | 102 |
| Minimum | 0 | 0 | 0 | 0 | 0 | 0 | 0 | 0 | 0 |
| 25% Percentile | 0.01216 | 0.05848 | 0.09111 | 0.0323 | 0.04275 | 0.05917 | 0.177 | 0.1646 | 0.09352 |
| Median | 0.1017 | 0.1539 | 0.1763 | 0.05885 | 0.07165 | 0.1009 | 0.2366 | 0.239 | 0.1361 |
| 75% Percentile | 0.2285 | 0.2716 | 0.3089 | 0.07956 | 0.0923 | 0.1489 | 0.3028 | 0.2964 | 0.1952 |
| Maximum | 0.5502 | 0.5405 | 0.6161 | 0.1655 | 0.1766 | 0.3788 | 0.5233 | 0.5009 | 0.6117 |
| Mean | 0.1445 | 0.1777 | 0.2077 | 0.05883 | 0.07019 | 0.1096 | 0.2405 | 0.2385 | 0.152 |
| Std. Deviation | 0.1459 | 0.1319 | 0.1462 | 0.03654 | 0.03939 | 0.06655 | 0.09659 | 0.09171 | 0.09079 |
| Std. Error of Mean | 0.009724 | 0.01171 | 0.01448 | 0.002436 | 0.003495 | 0.00659 | 0.006439 | 0.008138 | 0.00899 |
| Lower 95% CI of mean | 0.1253 | 0.1545 | 0.179 | 0.05403 | 0.06327 | 0.09655 | 0.2278 | 0.2224 | 0.1341 |
| Upper 95% CI of mean | 0.1636 | 0.2009 | 0.2364 | 0.06363 | 0.07711 | 0.1227 | 0.2532 | 0.2546 | 0.1698 |
| Sum | 32.5 | 22.57 | 21.18 | 13.24 | 8.914 | 11.18 | 54.12 | 30.29 | 15.5 |
| D'Agostino & Pearson normality test |  |  |  |  |  |  |  |  |  |
| K2 | 28.33 | 9.002 | 13.46 | 4.128 | 2.23 | 14.7 | 0.81 | 2.52 | 49.77 |
| P value | <0.0001 | 0.0111 | 0.0012 | 0.1269 | 0.328 | 0.0006 | 0.667 | 0.2837 | <0.0001 |
| Passed normality test (alpha=0.05)? | No | No | No | Yes | Yes | No | Yes | Yes | No |
| P value summary | **** | * | ** | ns | ns | *** | ns | ns | **** |
| Shapiro-Wilk normality test |  |  |  |  |  |  |  |  |  |
| W | 0.8723 | 0.9446 | 0.9144 | 0.9757 | 0.9819 | 0.9614 | 0.9933 | 0.9877 | 0.8919 |
| P value | <0.0001 | <0.0001 | <0.0001 | 0.0007 | 0.0874 | 0.0045 | 0.4131 | 0.316 | <0.0001 |
| Passed normality test (alpha=0.05)? | No | No | No | No | Yes | No | Yes | Yes | No |
| P value summary | **** | **** | **** | *** | ns | ** | ns | ns | **** |
| Actual mean | 0.1445 | 0.1777 | 0.2077 | 0.05883 | 0.07019 | 0.1096 | 0.2405 | 0.2385 | 0.152 |
| Discrepancy | 0.1445 | 0.1777 | 0.2077 | 0.05883 | 0.07019 | 0.1096 | 0.2405 | 0.2385 | 0.152 |
| 95% CI of discrepancy | 0.1253 to 0.1636 | 0.1545 to 0.2009 | 0.179 to 0.2364 | 0.05403 to 0.06363 | 0.06327 to 0.07711 | 0.09655 to 0.1227 | 0.2278 to 0.2532 | 0.2224 to 0.2546 | 0.1341 to 0.1698 |
| t, df | t=14.85 df=224 | t=15.18 df=126 | t=14.35 df=101 | t=24.15 df=224 | t=20.08 df=126 | t=16.64 df=101 | t=37.35 df=224 | t=29.31 df=126 | t=16.9 df=101 |
| P value (two tailed) | <0.0001 | <0.0001 | <0.0001 | <0.0001 | <0.0001 | <0.0001 | <0.0001 | <0.0001 | <0.0001 |
| Significant (alpha=0.05)? | Yes | Yes | Yes | Yes | Yes | Yes | Yes | Yes | Yes |
